# Arranging the Teeth on the Wax-Trial Plate

**Published:** 1906-11

**Authors:** Stewart J. Spence

**Affiliations:** Chattanooga, Tenn.


### ARRANGING THE TEETH ON THE WAX-TRIAL PLATE.

STEWART J. SPENCE, CHATTANOOGA, Tenn.

Having placed the models on the articulating frame, as described in my last previous paper, proceed to set up the teeth on wax on said models.

The first thing to decide is the amount of overbite. In nature this is usually about 1/8 inch, but in most porcelain teeth it is from 1-16 to 1-52; and therefore if the dentist desires to give the long cusps and deep occlusal groove that accompany a long bite it is usually necessary to grind the bicuspids and molars. An emery (or preferably, carborundum) wheel of about 1/2 inch thickness may, for this purpose, be given a knife edge at the periphery of its circumference by holding against it another carborundum wheel while revolving it on lathe. With this instrument it is best to cut the occlusal groove to proper depth before commencing to set up the teeth, making small changes afterwards if necessary. If the overbite at the incisors is 1/8 inch, it will at the second molar be 1-16 inch.

If this groove is of correct depth, it follows that the cusps must be of correct height; therefore, in setting up the teeth on the wax, if the cusps so interlock as to touch each other at all points in the grinding bite, they must of necessity occlude correctly in the groove.

Long, keen, cusps are much more efficient as masticators than blunt ones or cuspless teeth; therefore they ought to be used. It is a mistake to suppose that long cusps bend to dislodgement of plates. True, they do so if not correctly articulated, but if made to occlude properly in all the various relative positions of the jaws, they never interfere, and—on the contrary—can be made very serviceable in avoiding dislodgement.

This is done thus: Dislodgement, be it remembered, is more apt to occur in the grinding, or lateral, bite than in any other; not only because its sideward movement tends to displace the plate in the opposite direction from that in which the bite-motion is going, but also because this lateral bite is made about twenty times to one incising bite, and hundreds of times to one protrusion bite. By this latter I mean where incisors occlude tip to tip in making the incising bite, like nippers, instead of, as is usual, merely passing each other like the blades of a pair of shears. Therefore, because dislodgment is most apt to occur in the lateral bite, it is necessary to guard against this as much as possible by causing both sides of the denture to occlude simultaneously; that is, at the time the incisors, bicuspids and molars are coming in occlusion on one side, the cusps of the last molars should have come into occlusion on the other side of the mouth, so that by this equal pressure the plate is held in place.

This desirable simultaneous occlusion will always be present if the occlusal groove has been ground to correct depth, as before described, and if the last molar or two be given the correct upward incline of occlusal surface.

This incline is usually at an angle of about 25 degrees from the plane of the incising edges and buccal cusps of all the other anterior teeth. Thus if an upper set of porcelain teeth are correctly set up, they may be placed on a flat surface and their tips will be seen to each touch this surface until they come to the second molar, which rises from it at the obtuse angle above mentioned.

There is, however, a slight exception to this rule, viz., that the posterior palotal cusp of the first molar falls below this plane, being longer than its adjacent buccal cusp. This is important. Commencing at the first bicuspid, these palatal cusps gradually increase in height, being in the bicuspids shorter than the palatal cusps, and being about equal in the first molar (or rather, its second palatal cusp being usually a little longer), and longer in the second and third molars. As these long palatal cusps of these last molars articulate in the occlusal groove of the lower molars, said groove must be made shallower on the lingual side than the palatal. That is, the lingual cusps of the lower grinding teeth are all on a lower level than their buccal cusps. Were they not thus lower, they would occlude prematurely with the palatal cusps of the superior molars in the grinding bite.

Instead, therefore, of nature providing us with four rows of cusps of even height, they are arranged in the varied manner here described.

In setting up teeth, when it is desirable to slightly change the relative heights of the buccal and lingual cusps of save one tooth, this can usually be done better by simply tilting the tooth a little, buccally or lingually, instead of resorting to the slow process of regrinding it. Nature thus tilts the lower grinding teeth lingually, and the upper buccally.

If correct articulation is made, as here described, it is not a matter of much importance whether or not the teeth sit directly under the ridge. Indeed, to place them under the upper ridge after considerable absorption has occurred necessitates placing the opposing lower teeth away from the lower ridge into the region of the tongue, which is to be avoided, because the lower plate needs to be fitted much more than does the upper. Of course, where it is possible to keep the teeth under the ridge without doing so at the expense of anything else, it is very well to so do.

I am not an advocate of the popular teaching that the upper and lower incisors should not be allowed to touch; nor of the other theory, that the last molars should be given a little space between them and the burden of occlusion made to fall about the bicuspids. These rules are useful only when correct and perfect articulation is not attained; but when teeth are set up in nature's ideal method, as here described, these rules are needless.

Great care is necessary as regards correct bite. The teeth when set up should be tried in, and any error of bite detected; because to attempt to correct error by any extensive grinding after the plate is made is to destroy the "simultaneous bite" here insisted on. To this end, if any grinding of the incising surfaces of the anterior teeth is done, in order to give them a worn and unartificial appearance, this should not be left to be done when plate is inserted.

